# Emerging therapies in advanced hepatocellular carcinoma

**DOI:** 10.1186/s40164-018-0109-6

**Published:** 2018-08-03

**Authors:** Sowmini Medavaram, Yue Zhang

**Affiliations:** Stony Brook University Hospital, 101 Nicolls Road, Stony Brook, NY USA

**Keywords:** Advanced HCC, TKI, Immunotherapy, Chemotherapy

## Abstract

**Background:**

Prognosis is very poor for advanced HCC patients partially due to lack of effective systemic treatment. Sorafenib was the only approved agent for advanced HCC since 2007 until recent breakthroughs. In this article, we will review the newer approved and promising agents in the treatment of advanced HCC in the first line setting and beyond progression.

**Main body:**

The Food and Drug Administration approved sorafenib as it demonstrated 3 months overall survival benefit compared to placebo in the first line setting over 10 years ago. Multiple single agent and combination therapies have been studied but failed to show benefit. Chemotherapy has limited role in patients with advanced HCC given poor hepatic reserve due to underlying cirrhosis. A new era of treatment for advanced HCC arrived recently with exciting data presented for lenvatinib, regorafenib, cabozantinib, nivolumab, ramucirumab and several other promising clinical trials.

**Conclusion:**

Advanced HCC patients are difficult to treat with poor outcomes. After initial approval of sorafenib in 2007, we recently have multiple new agents that showed benefit and promising activity, and are set to change the landscape of HCC treatment.

## Background

Hepatocellular carcinoma (HCC) is the third leading cause of cancer death worldwide resulting in nearly 745,000 deaths each year [1]. Chronic Hepatitis B (CHB) infection is the etiology for maximum number of HCC diagnosis worldwide, particularly in Asia. Chronic Hepatitis C (HCV), alcoholic cirrhosis and non-alcoholic steatohepatitis (NASH) are the main causes in Western population. Incidence of HCC is increasing in the US over the past decade. Incidence and deaths from HCC were 40,710 and 28,920 in the US in 2017 approximately [2].

Treatment options for HCC were limited. The Barcelona clinic liver cancer (BCLC) staging system is established and widely used to guide HCC treatment options. Surgical resection and liver transplantation offer potential cure for HCC. Although surgical resection offers option for cure, there is a high chance of recurrence in the remaining liver in the setting of underlying cirrhosis [3]. On the otherhand, liver transplantation (LT) has the ability to remove tumor as well as underlying cirrhosis [4]. LT is limited by graft shortage and need for appropriate patient selection. Milan criteria (MC) are widely used to select patients with HCC for LT [5]. 10-year overall survival was 70% in 300 liver transplants for HCC that fulfilled the MC [6]. Prioritization of liver transplant candidates on waiting list, decreasing dropout rates and shortening the waiting time play an important role in better outcomes [7]. Local directive therapies are widely used in HCC such as radiofrequency ablation, radioembolization (Y90), transarterial chemoembolization (TACE), stereotactic body radiation therapy (SBRT). However, these treatments focus on local disease control as the main treatment benefit. Diagnosis of HCC is often made when the disease is advanced, given lack of symptoms in the early stage. Prognosis of advanced HCC remains very poor. Management of advanced HCC is particularly challenging because of poor liver function from underlying cirrhosis. Sorafenib gained the Food and Drug Administration (FDA) approval for HCC in 2007. It remained the only systemic treatment option for advanced HCC for almost a decade until recent progress [8, 9]. Child–Pugh score often dictates therapeutic options. Systemic treatment is usually not offered to patients with Child–Pugh C cirrhosis due to poor liver function and dismal prognosis from underlying cirrhosis. In a registry study of sorafenib in HCC patients, the median overall survival (OS) of HCC patients was 13.6 months, 5.2 months and 2.6 months respectively for Child–Pugh A, B and C patients [10]. Chemotherapy might have a potential role but with considerable risks. Development of new therapies for advanced HCC has made nearly no progress since the approval of sorafenib until recently. After failure of multiple tyrosine kinase inhibitors (TKI) in HCC in the last decade, Lenvatinib, cabozantinib and regorafenib were proven to have efficacy in HCC in most recent clinical trials [11–13]. Ramucirumab also demonstrated survival benefit in selected HCC patients [14]. Immunotherapy has been changing the landscape of oncology in recent years and appears very promising in HCC as well. Nivolumab obtained accelerated approval for second line HCC [15]. A number of exciting clinical trials are in progress currently, which are studying immunotherapy in HCC. In this article, we will review the potential systemic therapies for advanced HCC.

### First line treatments

Sorafenib is a small multi-tyrosine kinase inhibitor that blocks the activity of Raf kinase, vascular endothelial growth factor (VEGF) receptor and platelet-derived growth factor (PDGF) receptor. It was the first and only treatment of advanced HCC for over 10 years. It was approved in 2007 based on the results of Sorafenib HCC Assessment Randomized Protocol [SHARP] study [8, 9]. This study included 602 patients with preserved liver function and were randomly assigned to sorafenib 400 mg twice-daily vs placebo. Survival benefit was modest with median overall survival of 10.7 months vs 7.9 months in sorafenib vs placebo group (HR = 0.69; 95% confidence interval [CI] 0.55–0.87; p < 0.001). Response rate (RR) was very low with only seven patients in the sorafenib group (2%) for a partial response [9]. Sorafenib reports to be more beneficial in patients with HCV infected subgroup than HCC patients with other underlying etiologies. In the exploratory analysis of SHARP trial, HCV-infected patients treated with sorafenib compared with placebo had superior median overall survival (14.0 vs 7.4 months), compared to hepatitis B positive patients (9.7 vs 6.1 months) and alcohol-related HCC patients (10.3 vs 8.0 months), respectively [16]. Sorafenib has been associated with increased frequency of diarrhea, weight loss, hand–foot skin reaction, and hypophosphatemia [9]. Majority of the study patients included in the clinical trials were Child–Pugh A. Most of the oncologists dose modify for Child–Pugh B patients while do not even offer sorafenib to Child–Pugh C patients due to safety concerns. Regardless, sorafenib has remained the only approved systemic treatment for advanced HCC patients for almost a decade. There was a demanding need for improvement of HCC patient care.

Lenvatinib, an orally active, novel tyrosine-kinase inhibitor with multiple targets, including VEGFR 1–3, fibroblast growth factor receptor (FGFR 1–4), PDGFRα, RET, is the first breakthrough after multiple failures of other TKIs in the first line setting recently. A phase III randomized study (REFLECT trial) demonstrated non-inferiority of lenvatinib vs sorafenib in previously untreated unresectable HCC (overall survival OS: 13.6 vs 12.3 months; hazard ratio 0.92, 95% CI 0.79–1.06) [13]. A total of 954 eligible patients were randomized to receive oral lenvatinib (12 mg/day for bodyweight ≥ 60 kg or 8 mg/day for bodyweight < 60 kg) or sorafenib 400 mg twice-daily in 28 day cycles. Even though OS was similar between the agents, lenvatinib showed significant and clinically meaningful improvements in progression-free survival (PFS) over sorafenib (7.4 months vs 3.7 months), overall response rate (RR) (24% vs 9%) and time to progression (8.9 months vs 3.7 months) respectively [13]. The side effects observed for lenvatinib were hypertension, proteinuria, dysphonia and hypothyroidism as opposed to palmar-plantar erythrodysaesthesia and diarrhea with sorafenib. Use of modified RECIST criteria to assess response is a limitation of the study [13].

A number of other agents have been also investigated in the first line setting either alone (bevacizumab, sunitinib, brivanib) or in combination with sorafenib (doxorubicin, erlotinib) in the past [17–21]. Unfortunately, none of them showed significant improvement in survival over sorafenib alone [17–21]. Bevacizumab, monoclonal antibody directed against VEGF, given as single agent as 5 or 10 mg/kg every 2 weeks showed objective response rate of 13% with a PFS of 6.9 months [20]. Sunitinib, an oral multi- targeted tyrosine kinase inhibitor, showed inferior survival compared to sorafenib (7.9 vs 10.2 months) in a phase III trial, which enrolled 1073 previously untreated advanced HCC patients and had significantly worse adverse effects [18]. Combination of erlotinib and sorafenib showed no improved survival compared to sorafenib alone in a phase III trial [21]. Chemotherapy alone has unclear benefit in advanced HCC. Doxorubicin is the most commonly studied agent with response rates around 20% [22]. The phase III CALGB trial 80802 study failed to show the benefit of adding doxorubicin to sorafenib. The median OS with sorafenib monotherapy was 10.5 months vs 9 months with doxorubicin plus sorafenib in the Cancer and Leukemia Group B (CALGB) 80802 [17].

Both sorafenib and lenvatinib have shown benefit in the first line setting. Choosing the right agent for the appropriate patient becomes challenging. Although survival benefit was seen in all sub-groups in SHARP study, HCV infected patients treated with sorafenib had OS of 14 months vs 7.4 months whereas patients with HBV had OS of 9.7 months vs 6.1 months compared to placebo. This indicates that sorafenib has better outcomes in patients with HCV. OS was better in patients without macrovascular invasion and extra-hepatic spread with sorafenib. However, SHARP trial was not randomized by these sub-groups and were at a risk of imbalance [16]. Lenvatinib has showed better progression free survival, time to progression and response rate over sorafenib in REFLECT trial [13]. This might play an important role when deciding on treatment plan. Lenvatinib might be more attractive to be used in sub-set of patients with more extensive disease and for patients who are intolerant to sorafenib due to its side-effects. This has to be addressed in randomized trials before a conclusion can be made. Sorafenib has been used in the last 10 years and oncologists are more familiar with its toxicity profile and management. Lenvatinib still needs time to gain familiarity in day to day practice. A further understanding of the balance of quality of life and toxicity management will be important in determining their role in the future of HCC management [23]. Table 1 lists the selected agents that have been tested in the first line setting.

**Table 1:** Selected agents in first line for advanced HCC

Trial	Phase	Mechanism of action	Primary endpoint	OS (months)	PFS (months)	ORR (%)
Sorafenib vs placebo (SHARP**)**	III	Multi-targeted TKI	OS	10.7 vs 7.9 months (p < 0.001)	5.5 vs 2.8 months (p < 0.001)	2
Lenvatinib vs sorafenib (REFLECT)	III	Multi-targeted TKI	OS, non-inferiority	13.6 vs 12.3 months (HR = 0.92; 95% CI 0.79–1.06)	8.9 vs 3.7 months (p < 0.0001)	24.1 vs 9.2 (p < 0.0001)
Sunitinib vs sorafenib	III	Multi-targeted TKI	OS	7.9 vs 10.2 months (p = 0.0014)	3.6 vs 3.0 months (p = 0.22)	–
Sorafenib + erlotinib vs sorafenib + placebo	III	EGFR TKI	OS	9.5 vs 8.5 months (p = 0.408)	3.2 vs 4 months (p = 0.18)	6.6 vs 3.9 (p = 0.102)
Bevacizumab + Erlotinib	II	EGFR TKI Anti-VEGF	PFS	13.7 months	7.2 months	24
Sorafenib + doxorubicin vs sorafenib alone	III	Multi-targeted TKI Cytotoxic agent	OS	9.3 vs 10.5 months	3.6 vs 3.2 months	–
Bevacizumab	II	Anti-VEGF	PFS	–	6.9 months	13
Ramicirumab	II	Anti-VEGF	PFS	12 months	4 months	9.5

*TKI* tyrosine kinase inhibitor, *EGFR* epidermal growth factor receptor, *VEGFR* vascular endothelial growth factor, *OS* overall survival, *PFS* progression free survival

### Second line treatments

#### Targeted therapies

Sorafenib was the only approved agent for advanced HCC for a long time. For patients who have progressed or intolerant to sorafenib, there were no standard of care. Chemotherapy has been tried with unclear role in this setting and most of the patients go to hospice care directly. There was an urgent unmet need for almost a decade until recent breakthroughs. FDA recently approved regorafenib and nivolumab as second therapy after progression on sorafenib [12, 15]. Cabozantinib has also showed overall survival benefit in a recent phase III study [11]. Ramucirumab demonstrated survival benefit as well in patients with elevated alpha-fetoprotein (AFP) > 400 ng/ml at diagnosis [14].

Regorafenib is the agent approved for second line HCC in April 2017, based on the results of phase III, randomized, double-blind, and placebo controlled trial (RESORCE) [12]. Regorafenib is an orally active inhibitor of angiogenic VEGF1-3, oncogenic and stromal receptor tyrosine kinases. The study selected patients who tolerated sorafenib (≥ 400 mg daily for at least 20 of the 28 days before discontinuation) and had Child–Pugh A liver function. A total of 573 patients were randomized with regorafenib showing improved OS (10.6 months vs 7.8 months for placebo) with a hazard ratio of 0.63 (95% CI 0.50–0.79; one-sided p < 0.0001) [12]. The most common grade 3 or 4 adverse events included hypertension, hand–foot skin reaction, fatigue, and diarrhea [12]. Since this study only included patients who progressed while on sorafenib and required a minimum of 20 out of 28 days of at least 400 mg daily dose, overall survival benefit conclusions about the efficacy of regorafenib in patients who do not tolerate sorafenib cannot be drawn. This study also showed sequential use of multikinase inhibitors with appropriate management of adverse events led to extension in survival [23].

Cabozantinib, an inhibitor of MET, VEGFR, and AXL, was studied in double-blind, randomized, global, phase 3 trial (CELESTIAL trial) compared to placebo in previously treated patients with advanced HCC [11]. However, previous tolerance to sorafenib was not required. Primary outcome was overall survival. A total of 707 patients were randomized and the results showed median OS 10.2 months for Cabozantinib vs 8.0 months for placebo (HR 0.76, 95% CI 0.63–0.92; p = 0.0049). Median PFS was statistically improved as well from 5.2 months vs 1.9 months (HR 0.44, 95% CI 0.36–0.52; p < 0.0001) [11]. Hand-foot skin reaction (17% vs 0%), hypertension (16% vs 2%), increased aspartate aminotransferase (12% vs 7%), fatigue (10% vs 4%), and diarrhea (10% vs 2%) were reported in cabozantinib vs placebo group [11]. Due to the significant improvement of OS and PFS, cabozantinib can potentially gain FDA approval in the second line setting of advanced HCC.

Ramucirumab is a monoclonal antibody binds to VEGFR-2 receptor and block its activation. Initial phase II trial of 42 patients showed ORR of 10% and median OS of 12 months indicating modest degree of activity in patients with no prior systemic treatment [24]. A phase III trial of ramucirumab compared to placebo in patients with advanced HCC following first-line treatment with sorafenib (REACH) failed to show a significant survival advantage (median OS 9.2 vs 7.6 months) [25]. However, unplanned subset analysis suggested survival benefit in patients with elevated AFP > 400 ng/ml at diagnosis [25]. A follow up phase III trial (REACH-2) in 292 patients with baseline elevated AFP was conducted and confirmed significant survival benefit of ramucirumab treatment in patients who progressed or were intolerant to sorafenib [14]. Ramucirumab has significantly improved OS: 8.5 months vs 7.3 months (p = 0.0199); PFS: 2.8 months vs 1.6 months (p < 0.0001); and disease control rate: 59.9% vs 38.9% (p = 0.0006) compared to placebo [14]. Hypertension and hyponatremia were observed more in the study arm for serious toxicities [14]. Patients with elevated AFP at baseline have worse prognosis in general. REACH-2 is the first positive study in this particular biomarker selected patient population.

Everolimus, mammalian target of rapamycin (mTOR) inhibitor, was studied in a large phase III trial of patients with progression or intolerance to sorafenib [26]. mTOR is a key regulator of cell growth and angiogenesis and this pathway is activated in 40–50% of patients with HCC. This study showed no improvement in OS (7.6 vs 7.3 months with everolimus and placebo respectively) [26].

Axitinib is a selective second generation TKI that targets the VEGFRs. It was studied in a phase II trial compared to best supportive care in 202 patients previously treated or intolerant to sorafenib with advanced HCC. The difference in median OS (12.7 vs 9.7 months) was not statistically significant [27]. Its use cannot be recommended outside of clinical trial.

Brivanib is a selective dual inhibitor of fibroblast growth factor (FGF) and VEGF receptor tyrosine kinases. FGF proteins are involved in tumor growth and angiogenesis in HCC. Phase III BRISK-PS trial evaluated the efficacy and safety of brivanib in patients with advanced HCC who had disease progression on or intolerance to sorafenib. There was no significant improvement in the median OS compared to placebo (9.4 vs 8.2 months, HR = 0.89; 95.8% CI 0.69–1.15; p = 0.3307) [28]. Table 2 lists the various studies done in the second setting line setting.

**Table 2:** Selected clinical trial for advanced HCC in the second line

Trial	Phase	Mechanism of action	Primary endpoint	OS (months)	PFS (months)	ORR (%)
Regorafenib vs placebo (RESORCE)	III	Multi-targeted TKI	OS	10.6 vs 7.8 months (p < 0.0001)	3.1 vs 1.5 months (p < 0.0001)	11 vs 4 (p = 0.0047)
Cabozantinib vs placebo (CELESTIAL**)**	III	Multi-targeted TKI	OS	10.2 vs 8.0 months (p = 0.0049)	5.2 vs 1.9 months (p < 0.001)	4 vs 0.4 (p = 0.0086)
Nivolumab	I/II	PD-1 immune checkpoint inhibitor	ORR	15 months	–	20 (95% CI = 15–26)
Pembrolizumab	II	PD-1 immune checkpoint inhibitor	ORR	–	4.8 months	16.3
Ramucirumab vs placebo	III	Anti-VEGF	OS	9.2 vs 7.6 months (p = 0.14)	2.8 vs 2.1 months (p < 0.0001)	7 vs < 1 (p < 0.0001)
Axitinib + BSC vs placebo + BSC	II	Anti-VEGF	Tumor control	12. 7 vs 9.7 months (p = 0.287)	3.6 vs 1.9 months (p = 0.004)	9.7 vs 2.9 (p = 0.091)
Brivanib vs placebo	III	FGFR inhibitor	OS	9.4 vs 8.2 months (p = 0.3307)	4.2 vs 2.7 months (p < 0.001)	10 vs 2
Everolimus vs placebo	III	mTOR inhibitor	OS	7.6 vs 7.3 months (p = 0.68)	3 vs 2.6 months	8 vs 3

*TKI* tyrosine kinase inhibitor, *PD-1* programmed cell death protein-1, *VEGF* vascular endothelial growth factor, *FGFR* fibroblast growth factor receptor, *mTOR* mechanistic target of rapamycin, *OS* overall survival, *ORR* objective response rate, *PFS* progression free survival

#### Immunotherapy

Immunotherapy is changing the landscape of oncology care in the last few years. Several immunotherapies, such as tumor antigen therapy, immune checkpoint inhibitors, and adoptive cell transfer (ACT) immunotherapy are currently being studied in HCC [29]. Multiple checkpoint inhibitors have gained FDA approval for many different types of malignancies [30, 31]. It has also been studied in several gastrointestinal malignancies with meaningful clinical activity [32]. Several exciting clinical trials for advanced HCC patients are currently ongoing.

Nivolumab, an immune checkpoint inhibitor, was studied in a phase I/II multi-cohort trial (checkmate 040). It included a total of 262 eligible patients with HCC and Child–Pugh A cirrhosis who are either sorafenib naïve or had progression on sorafenib [31]. Dose escalation phase established the safety profile of 3 mg/kg dose. Nivolumab 3 mg/kg was given every 2 weeks in the dose-expansion phase and showed objective response rate of 15–20% irrespective of line of therapy [15]. Responses occurred within 3 months in 69% of responders. This study also showed 18 month-OS rates of 57% in sorafenib naïve and 44% in sorafenib experienced patients. Responses occurred regardless of HCC etiology or tumor cell programmed death-ligand 1 (PD-L1) expression [33]. The common adverse reactions were similar to the previously reports for nivolumab with a higher incidence of elevations in transaminases and bilirubin levels. Immune-mediated hepatitis requiring systemic corticosteroids occurred in 8 (5%) patients [34]. Nivolumab was granted accelerated approval based on durable response rates. Previous tolerance to sorafenib and rapidity of progression of disease might be taken into consideration of choosing regorafenib vs nivolumab in this setting. Nivolumab is currently being evaluated in Check Mate 459 phase 3 trial (NCT02576509) in comparison to sorafenib as a first- line treatment in patients with advanced HCC.

Pembrolizumab is another checkpoint inhibitor. It was studied in a single arm, open-label phase II KEYNOTE-224 study. 105 patients were enrolled with objective response rate of 16.3% (95% CI 9.8–24.9%), with 1 complete response. Median PFS was 4.8 months and median OS was not reached. The 6 month PFS and OS rates were 43.1% and 77.9%, respectively [35]. The phase III KEYNOTE-240 is currently underway assessing the efficacy of pembrolizumab for pre-treated patients with HCC (NCT02702401).

Another check point inhibitor anti cytotoxic T-lymphocyte-associated protein 4 (CTLA-4) antibody tremelimumab is studied in Advanced HCC. It was tested in a pilot clinical trial (NCT01008358) in patients with HCC and HCV infection. Disease control rate was 76.4% and time to tumor progression was 6.5 months [36]. Even though immunotherapy has achieved impressive results, a considerable proportion of patients do not respond. It has become important to identify patients who might respond to immunotherapy, thus paving a path for precision immunotherapy. It likely depends on number of factors such as tumor mutation burden, microsatellite instability status, neoantigen intratumoral heterogeneity, and immunohistochemistry biomarker assay for PDL-1 expression [31, 37, 38]. Table 3 lists the selected ongoing clinical trials with immunotherapy.

**Table 3:** Selected clinical trial with immunotherapy for advanced HCC

NCT number	Treatment	Target	Outcome	Phase
NCT02702401	Pembrolizumab vs best supportive care	PD-1	PFS, OS	Phase III
NCT02576509	Nivolumab vs sorafenib	PD-1	OS	Phase III
NCT02519348	Durvalumab + tremelimumab vs durvalumab monotherapy	PD-L1/CTLA-4	Adverse events	Phase II
NCT03298451	Durvalumab vs durvalumab + tremelimumab vs sorafenib HIMALAYA	PD-L1/CTLA-4	OS	Phase III
NCT01658878	Nivolumab vs nivolumab + ipilimumab vs sorafenib	PD-L1/CTLA-4	ORR	Phase I/II

*PD-1* programmed cell death protein-1, *PD-L1* programmed death-ligand 1, *CTLA-4* cytotoxic T-lymphocyte-associated protein-4, *OS* overall survival, *PFS* progression free survival, *ORR* overall response rate

Atezolizumab, anti-PD-L1 agent was studied in combination with bevacizumab in a phase Ib study in previously untreated patients with unresectable or metastatic HCC [39]. Patients received atezolizumab 1200 mg + bevacizumab 15 mg/kg every 3 weeks until progression or unacceptable toxicity. Primary end point was the safety and tolerability of the combination. At a median follow-up of 10.3 months, ORR was 65% among 23 evaluable patients. Most common treatment related grade 3–4 adverse event was hypertension. FDA granted a breakthrough therapy designation for the combination as a first-line treatment for patients with advanced or metastatic HCC. The ongoing phase III IMbrave150 study (NCT03434379) is comparing atezolizumab/bevacizumab with sorafenib in the frontline setting for patients with locally advanced or metastatic HCC.

Chimeric antigenic receptor T cell (CAR-T) is a form of ACT Immunotherapy. It has been a huge success in treating refractory hematological malignancies, most notably B-cell acute lymphoblastic leukemia [40]. Tumor cells express many aberrant proteins/antigens on surface which can be used as markers to target them with genetically engineered T cells [41]. Expanding CAR-T therapy to solid tumors is very attractive but has many challenges. Identifying the precise target antigen and designing CARs that are highly selective are critical for clinical application of such therapies. Many studies are ongoing to explore the possibility of CAR-T immunotherapy in HCC and other solid tumors [42, 43]. AFP is one such antigen which can be used as a specific target for CAR-T therapy in HCC [42]. AFP is a secreted glycoprotein that is commonly overexpressed in tumors of endodermal origin including pediatric hepatoblastoma and HCC. AFP has been reported to promote cell proliferation and suppress apoptosis suggesting its role in tumor progression [44, 45]. Other potential tumor associated antigens (TAA) in HCC are CEA, MUC1, MAGE-A1, NY-ESO-1, epithelial cell adhesion molecule (EPCAM) and Heat Shock Protein 70 (HSP70). Although CAR-T therapy appears very promising, it comes with many obstacles. Insufficient localization/penetration of CAR-T cells into tumor sites [46] and immunosuppressive tumor microenvironment have been implicated in the low efficacy [47, 48]. Lack of tumor specific antigens has proved to be a serious risk factor for toxicity associated with off-target or on-target/off-tumor effects [49]. Potential serious cytokine release syndrome (CRS) is another major toxicity associated with CAR-T therapy. Many of these obstacles can be controlled or removed by further progress. Specificity of CAR-T cells can be enhanced by tandem CAR, a novel bispecific CAR [50]. Poor localization and infiltration of CAR-T cells into solid tumors can be enhanced by expressing functional chemokine receptors like chemokine receptor type 2 (CCR2) [51]. Utilization of glucocorticoids and monoclonal antibody like tocilizumab which targets IL-6 receptors can help with treatment of CRS [52].

### Other options

#### Chemotherapy

Traditional cytotoxic chemotherapies such as capecitabine, oxaliplatin, cisplatin, fluoropyrimidine (5-FU or capecitabine), doxorubicin were studied in advanced HCC. Chemotherapy demonstrated limited efficacy [53–59]. Combination therapies showed response rates around 20% [54–56].Use of chemotherapy is a category 2B recommendation as per NCCN guidelines although they are not commonly used in daily practice. With the new data for TKIs and immunotherapy, its role in advanced HCC is less clear.

#### Combination therapy

TACE plus external beam radiotherapy (RT) showed benefit over sorafenib in previously untreated patients with macroscopic vascular invasion in an Asian clinical trial [60]. Ninety patients with liver target lesions and no extrahepatic metastasis were randomized to two groups. By intention to treat analysis, 12-week PFS (86.7% vs 34.3% at 12 weeks), RR (33.3% vs 2.2% at 24 weeks), and OS (55.0 vs 43.0 weeks) are all favor TACE-RT group over sorafenib group. Curative surgical resection was conducted for five patients (11.1%) in the TACE-RT group owing to downstaging [60]. This local therapy defines its potential role in management of selected advanced HCC patients.

TACE with sorafenib combination were studied in multiple previous studies for advanced HCC with promising results [61]. However, TACE with sorafenib did not improve PFS vs TACE with placebo in an European phase III trial recently [62]. It enrolled 399 patients with unresectable but liver-confined HCC. There was no evidence of difference in progression-free survival between the sorafenib group and the placebo group (HR 0.99 [95% CI 0.77–1.27], p = 0.94); median PFS was 238.0 days (95% CI 221.0–281.0) in the sorafenib group and 235.0 days (209.0–322.0) in the placebo group [62].

With the overall survival benefit with newer drugs for advanced HCC, we are waiting for approval of their labels for advanced HCC. The sequencing of these treatments will become more complicated. It will depend on the availability of medication, previous tolerance to sorafenib, oncologist’s experience, patient comorbidities and medication toxicity profile. Factors such as hepatitis status, disease burden, urgency of response, medication cost are all important. AFP is not an ideal biomarker but clearly showed utility in patient selection for ramucirumab.

### Future directives

Several combination therapies were discussed above without much success [60, 61]. However, with better understanding of the mechanism of action and management of toxicities, there is still a role of combination of local therapy with systemic treatment, or combination of different systemic treatments. Chemotherapy with immunotherapy combination showed benefit in lung cancer [63]. It needs further investigation in advanced HCC.

AFP has been widely used as the tumor marker for HCC but does not differentiate the complex etiology of HCC. It was used as a potential biomarker to direct treatment in the above mentioned REACH2 trial. However, further effort to explore biomarkers in advanced HCC to help understand the prognostic outcome and direct treatment options for different etiology are needed.

Circulating tumor cells (CTC) are emerging as biomarkers of the metastatic disease process, in addition to helping us understand tumor biology and tumor cell dissemination. CTC play a role in recurrence after undergoing LT for treatment of HCC. CTC hold a great promise in identifying patients at risk for relapse, stratification of patients to specific adjuvant therapies, and monitoring of response to treatment [64–66].

## Conclusions

Landscape in the management of Advanced HCC is changing rapidly over the past few years. In the first line setting, lenvatinib was non-inferior to sorafenib in a phase III trial, serving as an alternative to sorafenib which was the only systemic treatment available over the past decade. Recently, combination of atezolizumab and bevacizumab was given a breakthrough therapy designation by FDA for front line setting. Second line therapies have made rapid progress with approval of regorafenib in patients who tolerated sorafenib prior. Phase 3 trials of cabozantinib and ramucirumab has demonstrated survival benefit in sorafenib-experienced patients. Their approval will add more options to treatment armamentarium for advanced HCC. Immunotherapy has been making rapid progress with approval of nivolumab based on durable response rates. Various clinical trials are now underway to assess the role of immunotherapy in the first line setting. CAR-T therapy is under investigation and is making slow but a sure progress.

## Abbreviations

HCC: hepatocellular carcinoma
CHB: Chronic Hepatitis B
HCV: Chronic Hepatitis C
NASH: non-alcoholic steatohepatitis
BCLC: Barcelona clinic liver cancer
LT: liver transplantation
MC: Milan criteria
TACE: trans-arterial chemoembolization
SBRT: stereotactic body radiation therapy
FDA: Food and Drug Administration
OS: overall survival
TKI: tyrosine kinase inhibitors
VEGFR: vascular endothelial growth factor receptor
RR: response rate
PDGF: platelet derived growth factor
FGFR: fibroblast growth factor receptor
PFS: progression free survival
ORR: objective response rate
RT: radiotherapy
AFP: alpha fetoprotein
mTOR: mammalian target of rapamycin
FGF: fibroblast growth factor
PD-L1: programmed death-ligand 1
PD-1: programmed cell death protein-1
CTLA-4: cytotoxic T-lymphocyte-associated protein 4
EGFR: epidermal growth factor receptor
CAR-T: chimeric antigen receptor-T
PS: performance status
BSC: best supportive care
ACT: adoptive cell transfer
CRS: cytokine release syndrome
CTC: circulating tumor cells
